# Prevalence and Impact of BK Polyomavirus in the Ureters of Kidney Donors: Research Letter

**DOI:** 10.1177/20543581231166478

**Published:** 2023-04-10

**Authors:** Chagnon Sarah-Jane, Yangfan Zhao, Lamontagne Bruno, Royal Virginie, Lamarche Caroline

**Affiliations:** 1 Department of Medicine, Université de Montréal, Québec, Canada; 2 OPTILAB Montreal, Centre Hospitalier de l’Université de Montréal, Québec, Canada; 3 Department of Pathology, Hôpital Maisonneuve-Rosemont, Université de Montréal, Québec, Canada; 4 Hopital Maisonneuve-Rosemont Research Center, Department of Medicine, Division of Nephrology, Université de Montréal, Québec, Canada

**Keywords:** BK polyomavirus, transplant, qPCR, nephropathy, viremia

## Abstract

**Background:**

More than 75% of the population is seropositive for BK polyomavirus (BKV), which remains quiescent in the urothelium in immunocompetent hosts. However, it can reactivate in kidney transplant recipients (KTRs), and up to 30% of them will develop BKV viremia in the 2 years following transplant, with a risk of developing BKV-associated nephropathy (BKVAN). Viral reactivation is associated with the level of immunosuppression, but there is currently no way to predict which patients are at high risk for reactivation.

**Objective:**

As BKV originates from kidney donors, our primary objective was to determine the prevalence of detectable BKV in donor ureters. Our secondary objective was to see if there is a correlation between the presence of BKV in donor urothelium and the development of BKV viremia and BKVAN in KTR.

**Design:**

Prospective cohort study.

**Setting:**

Single-center academic kidney transplant program.

**Patients:**

Prospective sequential KTRs that received a kidney transplant between March 2016 and March 2017.

**Measurements:**

The presence of BKV in donor ureters was determined by TaqMan-based quantitative polymerase chain reaction (PCR; qPCR).

**Methods:**

We performed a prospective study which was done on 35 out of the 100 donors initially foreseen to take part in the study. During surgery, the distal part of donor ureter was kept and analyzed by qPCR (to establish the presence of BKV in the urothelium). The primary outcome was the development of BKV viremia in KTR over a period of 2 years after transplant. Secondary outcome was the development of BKVAN.

**Results:**

Out of 35 ureters analyzed, only one had a positive qPCR for BKV (2.86%, 95% confidence interval [CI]: [0.07-14.92]). Considering the primary objective would not be met, the study was interrupted after 35 specimens. After surgery, 9 recipients had a slow graft function and 4 had delayed graft function, one of which never recovered graft function. Over the 2-year follow-up, 13 patients developed BKV viremia, while 5 patients developed BKVAN. The patient who received a graft from a positive qPCR donor eventually developed BKV viremia and nephropathy.

**Limitations:**

The specimen analyzed was a distal rather than a proximal portion of the ureter. However, BKV replication is known to concentrate in the corticomedullary junction.

**Conclusion:**

BK polyomavirus prevalence in the distal part of donor ureters is lower than previously reported. It cannot be used as a predictor for the development of BKV reactivation and/or nephropathy.

## Abrege

**Contexte:**

Plus de 75 % de la population générale est séropositive pour le polyomavirus BK (BKV), qui demeure quiescent dans l’urothélium des hôtes immunocompétents. Il peut cependant se réactiver chez les receveurs d’une greffe rénale et, chez jusqu’à 30 % d’entre eux, entraîner une virémie BKV dans les deux ans suivant la transplantation, avec un risque de développer une néphropathie associée à BKV (NA-BKV). La réactivation virale est associée au niveau d’immunosuppression, mais il n’existe actuellement aucun moyen de déterminer quels patients présentent un risque élevé de réactivation.

**Objectifs:**

Puisque le BKV provient des donneurs de rein, l’objectif principal était d’établir la prévalence du BKV détectable dans les uretères des donneurs. L’objectif secondaire était d’examiner s’il existe une corrélation entre la présence de BKV dans l’urothélium du donneur et le développement d’une virémie BKV et d’une néphropathie associée au BKV chez les greffés rénaux.

**Type d’étude:**

Étude de cohorte prospective.

**Cadre:**

Programme de transplantation rénale d’un centre hospitalier universitaire.

**Sujets:**

Les receveurs séquentiels d’une greffe rénale qui avaient subi leur intervention entre mars 2016 et mars 2017.

**Mesures:**

La présence de BKV dans les uretères des donneurs a été déterminée par PCR quantitatif (qPCR) avec sonde TaqMan.

**Méthodologie:**

Notre étude prospective porte sur 35 des 100 donneurs initialement envisagés pour participer à l’étude. Pendant l’intervention, la partie distale de l’uretère du donneur a été conservée et analysée par qPCR (pour établir la présence de BKV dans l’urothélium). Le principal critère d’évaluation était le développement d’une virémie BKV chez les greffés rénaux dans les deux ans suivant la transplantation. Le résultat secondaire était le développement d’une néphropathie associée au BKV.

**Résultats:**

Sur les 35 uretères analysés, un seul s’est avéré positif pour BKV par qPCR (2,86 %; IC 95 %: 0,07-14,92). Voyant que l’objectif principal ne serait pas atteint, l’étude a été interrompue après 35 échantillons. Après la greffe, 9 receveurs ont expérimenté une lente reprise de la fonction du greffon et 4 receveurs ont vu la fonction de leur greffon retardée; de ces derniers, une personne n’a jamais retrouvé de fonction du greffon. Pendant les deux années de suivi, 13 patients ont développé une virémie BKV et 5 patients ont développé une néphropathie associée au BKV. La personne qui avait reçu le greffon du donneur positif au qPCR a ultérieurement développé une virémie et une néphropathie associées à BKV.

**Limites:**

Les échantillons analysés provenaient de la partie distale et non de la partie proximale de l’uretère. On sait toutefois que la réplication du BKV se concentre dans la jonction corticomédullaire.

**Conclusion:**

La prévalence du BKV dans la partie distale des uretères des donneurs s’est avérée plus faible que ce qui avait été précédemment rapporté. Ce résultat ne peut être utilisé comme prédicteur de la réactivation du BKV et/ou du développement d’une néphropathie associée à BKV.

## Introduction

BK polyomavirus (BKV) is a human-to-human acquired infection thought to be transmitted from the respiratory tract.^1^ An estimated 75% of the population are thought to be carriers for the virus,^2^ which remains in latency in the immunocompetent host.^3^ Host–virus interactions dictate a broad range of complications, from hemorrhagic cystitis in stem cell recipients^4^ to ureteral stenosis and nephropathy (BKVAN) in kidney transplant recipients (KTRs).^5^

The development of viremia and BKVAN is hypothesized to be the result of a 2-hit phenomenon. First, there is an increase in viral replication after transplant secondary to tubular cell injury that occurs through either ischemia-reperfusion or surgical trauma and the subsequent inflammatory response. Then, the intensity of immunosuppression regimens determines the timing and intensity of the viral reactivation (reviewed in)^3^.

To this day, the cornerstone of treatment remains the reduction of immunosuppression, putting patients at risk for graft rejection. Since no prediction model has proven to be effective in identifying patients at high risk for BKV reactivation, no preemptive conduct in the management of immunosuppression is currently in place before appearance of viremia.

After kidney transplantation, BKV is thought to be donor-derived. Indeed, Bohl and colleagues showed a concordance in viral genome in receiving pairs from the same donors.^6^ The only existing data on the prevalence of BKV in the urothelium originate from a study of Chesters in 1983 which was issued from 30 healthy donors.^7^ They found viral DNA in 33% of the kidneys using a hybridization technique. Considering BKV comes essentially from the donor and about 30% of recipients will develop BKV viremia,^8^ we hypothesized that the prevalence of this virus in the urothelium of renal grafts could be a reliable predictive factor for viremia development with relevance to risk stratification in transplant recipients. Therefore, we evaluated the prevalence of BKV in donor urothelium using current technical standards that are easily accessible at the time of transplant. We then evaluated the correlation between the presence of polyomavirus in donor kidneys and the development of viremia and BKVAN post-transplant.

## Methods

### Trial Design and Oversight

This is a prospective study conducted in a single transplant center. We evaluated the presence of BKV in the distal ureter of kidney donors by qPCR and correlated the results with clinical outcome (development of BKV viremia and nephropathy) in KTR. qPCR was used as is it sensitive, specific and has been used to detect BKV in tissue (tumor).^9^ Viremia was defined as more than 100 copies/mL which is our lower limit of detection. Biopsy-proven BKVAN was defined by a positive immunohistochemical staining for Simian Virus 40 (SV40) with compatible cytopathic changes. The study was approved by our institution’s Research Ethics Committee, in agreement with the Declaration of Helsinki.

### Participants and Data Collection

Patients who underwent a kidney transplant at Maisonneuve-Rosemont Hospital between March 2016 and March 2017 and who consented to the study were eligible. Collected data included age and sex of both donor and recipient, type of transplant donation (deceased or living), number of HLA incompatibilities, medication received for induction and maintenance therapy, cold ischemia time, presence of initial graft dysfunction, polyomavirus viremia development in the first 2 years after graft, and biopsy-proven BKVAN.

The follow-up is in agreement with the KDIGO recommendations and is standard practice in Maisonneuve-Rosemont Hospital since June 2010. We monitored the emergence of viremia monthly for the first 6 months, once every 2 months until the end of the first year, and then once every 3 months until the end of the second-year post-transplant. In the event of viremia development, the antimetabolite was reduced before the calcineurin inhibitor. A kidney biopsy was obtained for every patient with 2 BKV viremia of more than 10 000 copies/mL and/or clinical indication such as an increase in creatinine. Delayed graft function (DGF) was defined as the need for dialysis in the first week after transplant. Slow graft function was defined as less than 10% drop in creatinine per day for 3 consecutive days in the first week after transplant. Initial immunosuppression included 720 mg of mycophenolate acid twice a day, prednisone and a calcineurin inhibitor.

### Procedure

A frozen specimen of the donor’s distal urothelium was obtained at the moment of transplantation. A total of 35 fresh-frozen tissue samples were obtained and sent to the molecular diagnostic laboratory. Genomic DNA was extracted manually as follows: briefly, the samples were incubated in a 1.5 mL tube containing 200 µL extraction buffer (50 mM Tris pH 7.5, 50 mM NaCl, 10 mM ethylenediaminetetraacetic acid [EDTA] pH 7.5, and 0.005% sodium dodecyl sulfate [SDS]), and 100 µg/mL proteinase K. The tubes were mixed gently and incubated overnight at 56°C. If the samples were not fully digested, 5 µl of 20 mg/mL proteinase K were added and incubated for an additional hour at 56°C. After complete digestion, the 1.5 mL tubes were incubated at 90°C for an hour. Each tube was subjected to DNA extraction using phenol-chloroform and precipitated using 95% ETOH/2% KOAc. Each pellet was eluted in 32 μL H_2_O. A TaqMan-based quantitative PCR (qPCR) was then performed as previously described.^10^ A positive and a negative control were run every qPCR round.

### Efficacy Assessments

Primary outcome was the development of BKV viremia over a period of 2 years after transplant. Secondary outcome was the development of BKVAN.

### Statistical Analysis

We estimated that 100 donors and allograft recipients were required in order to have a power of 80%. However, the study was prematurely interrupted as only one out of the first 35 specimens analyzed had a positive result for BKV. Results were presented as percentages of the total number of specimens analyzed.

## Results

### Patient characteristics

We analyzed 35 transplant recipients from 35 different donors. Out of the 35 patients in which we analyzed the donor ureter, mean recipient age was 53.5 years, with a corresponding mean donor age of 47.7 years. Following transplant, 9/35 (26%) patients had a slow graft function, while 4/35 (11%) developed DGF, one of which never recovered graft function (Table 1). From the cohort studied, 6 (17%) donors were aged older than 60. Only 5 (14%) out of 35 donors had ≤ 2/6 mismatch when considering antigens A, B, and DR. Out of them, 3 (60%) patients eventually developed viremia, and 1 (20%) developed nephropathy. Cold ischemia time was more than 12 hours in 13 (37%) patients. Of these patients, 3 (23%) eventually developed DGF, 1 of which never regained graft function, and 3 (23%) had slow graft function.

**Table 1.** Patient Characteristics.

		Total	
Variable	No. (%) of patients n = 35	No. (%) of patients without BKV viremia, n = 22	No. (%) of patients with BKV viremia n = 13
Recipient age, yr: mean +/- SD (median, range)	53.5 +/-13.4 (54, 22-75)	52.4 +/- 13.2 (53, 22-70)	55.5 +/- 14.2 (57, 25-75)
Donor age, yr: mean +/- SD (median, range)	47.7 +/- 18.5 (52, 2-86)	45.1 +/- 18.2 (51.5, 2-73)	52.2 +/- 18.8 (53, 26-86)
HLA incompatibility in A, B, C and DR; mean +/- SD (median, range)	4.4 +/-1.8 (5, 0-8)	5.3 +/- 1.3 (5, 3-7)	4.5 +/- 1.4 (5, 0-8)
Cold ischemia time—hours; mean +/- SD (median, range)	10.3 +/- 6.2 (8.1, 2.1-23.8)	9.9 +/-5.5 (8.1, 2,7-21)	11.8 +/- 7.3 (10.3, 2.7-23.8)
Induction therapy Thymoglobuline Basiliximab	5 (14.3) 30 (85.7)	3 (13.6) 19 (86.4)	2 (15.4) 11 (84.6)
Initial maintenance therapy Tacrolimus, MMF, prednisone Cyclosporin, MMF, prednisone	34 (97.1) 1 (2.9)	22 (100) 0 (0)	12 (92.3) 1 (7.7)
Delayed graft function	4 (11.4)	3 (13.6)	1 (7.7)
Slow graft function	9 (25.7)	6 (27.3)	3 (23.1)
Biopsy-proven BKVAN	5 (14.3)	0 (0)	5 (38.5)
Positive qPCR for BKV in the donor’s ureter	1 (2.9)	0 (0)	1 (7.7)
BKV viremia duration, months; mean +/- SD (median, range)	-	-	13.2 +/- 15.8 (4, 1-50)
Quantitative BKV viremia, copies/mL; mean +/-SD (median, range)	-	-	285 781 +/- 602 838 (21 100, 2720-2 140 000)

BKV = BK polyomavirus; BKVAN = BK virus associated nephropathy; MMF = mycophenolate mofetil; SD = standard deviation; HLA = human leucocyte antigen; yr = year; qPCT = quantitative PCR.

### BKV in Donor Ureters, Viremia and Nephropathy

Unfortunately, BKV was only detected in 1 donor’s ureter out of 35 (2.86%; 95% confidence interval [CI] = [0.07-14.92]). Out of all allograft recipients, 13/35 (37%) eventually developed BKV viremia, and 7/35 (20%) had a viremia ≥ 10^4^ copies/mL. Five patients out of 35 (14%) developed biopsy-proven BKVAN. The patient who received a kidney from a donor positive for BKV before the graft eventually developed both viremia and nephropathy. The total period of detectable viremia on follow-up was only 4 months, from July 2016 to October 2016, and, interestingly, the patient maximal viremia was only 6.18 x 10^3^, which is lower than that of other patients with BKVAN. However, creatinine values remained stable before and during viremia, at around 150-160 µmol/L, and is now around 120-130 µmol/L 6 years after transplant.

## Discussion

Out of the 35 specimens of distal donor ureters analyzed, only one was positive for BKV. Considering the low detection of BKV in the donor samples, the primary objective would not be met and led to the premature interruption of the study. It is thus difficult to draw any definite conclusions. These results differ drastically from the 1983 publication by Chesters and colleagues, which identified a prevalence of 33% in the urothelium.^7^ It is reasonable to think that the analysis of distal ureter specimens led to an underdetection of the virus. BK polyomavirus is known to have a patchy distribution in the urothelium of the kidney and seems to have a predilection for replicating in the corticomedullary junction. As for the sensitivity of our assay, BKV qPCR from frozen samples were not directly compared to the method used by Chesters and al. but is known to be sensitive, and more than immunohistochemistry.^9^ Besides, our incidence rate of BKV viremia (37%) was slightly higher than previously reported. This could be related to a higher sensitivity of the qPCR used, as it is not a standardized assay across transplant centers.^10^ Unfortunately, we did not have access to the 35 matching kidneys from the same donors and could not evaluate the concordance in matching ureter nor the development of BK viremia. We also do not perform procurement biopsies, which could have been used to evaluate the presence of BKV in the corticomedullary junction.

## Conclusion

BK polyomavirus prevalence in the distal part of donor ureters was lower than expected and is probably not representative of the presence of BKV in the transplanted organ. It is not a good predictor for the development of BKV reactivation and/or nephropathy.

## References

[1] Goudsmit J Wertheim-van Dillen P van Strien A van der Noordaa J. The role of BK virus in acute respiratory tract disease and the presence of BKV DNA in tonsils. J Med Virol. 1982;10(2):91-99. 6292361 10.1002/jmv.1890100203

[2] Gardner SD . Prevalence in England of antibody to human polyomavirus (B.k.). Br Med J. 1973;1(5845):77-78. 20791873 10.1136/bmj.1.5845.77 PMC1588750

[3] Lamarche C Orio J Collette S , et al. BK Polyomavirus and the Transplanted Kidney: immunopathology and Therapeutic Approaches. Transplantation. 2016;100(11):2276-2287. 27391196 10.1097/TP.0000000000001333 PMC5084638

[4] Arthur RR Shah KV Baust SJ Santos GW Saral R. Association of BK viruria with hemorrhagic cystitis in recipients of bone marrow transplants. N Engl J Med. 1986;315(4):230-234. 3014334 10.1056/NEJM198607243150405

[5] Coleman DV Mackenzie EF Gardner SD Poulding JM Amer B Russell WJ. Human polyomavirus (BK) infection and ureteric stenosis in renal allograft recipients. J Clin Pathol. 1978;31(4):338-347. 205555 10.1136/jcp.31.4.338 PMC1145271

[6] Bohl DL Storch GA Ryschkewitsch C , et al. Donor origin of BK virus in renal transplantation and role of HLA C7 in susceptibility to sustained BK viremia. Am J Transplant. 2005;5(9):2213-2221. 16095500 10.1111/j.1600-6143.2005.01000.x

[7] Chesters PM Heritage J McCance DJ. Persistence of DNA sequences of BK virus and JC virus in normal human tissues and in diseased tissues. J Infect Dis. 1983;147(4):676-684. 6302172 10.1093/infdis/147.4.676

[8] Qeska D Wong RBK Famure O , et al. Incidence, risk factors, outcomes, and clinical management of BK viremia in the modern era of kidney transplantation. Transpl Infect Dis. 2022;24(6):e13915. 10.1111/tid.13915 35899972

[9] Llewellyn MA Gordon NS Abbotts B , et al. Defining the frequency of human papillomavirus and polyomavirus infection in urothelial bladder tumours. Sci Rep. 2018;8(1):11290. 30050097 10.1038/s41598-018-29438-y PMC6062511

[10] Lamontagne B Girard N Boucher A Labbé AC. Improved detection and quantitation of human BK polyomavirus by PCR assay. J Clin Microbiol. 2011;49(7):2778. 10.1128/JCM.00449-11 PMC3147848 21719733

